# Genetic epidemiology of titin-truncating variants in the etiology of dilated cardiomyopathy

**DOI:** 10.1007/s12551-017-0265-7

**Published:** 2017-05-05

**Authors:** Ali M. Tabish, Valerio Azzimato, Aris Alexiadis, Byambajav Buyandelger, Ralph Knöll

**Affiliations:** 1 Integrated Cardio Metabolic Centre (ICMC), Karolinska Institutet, 141 57 Huddinge, Sweden; 2 AstraZeneca R&D Mölndal, R&D, Innovative Medicines & Early Development, Cardiovascular & Metabolic Diseases iMed, 431 83 Mölndal, Sweden

**Keywords:** Titin, Heart failure, Dilated cardiomyopathy, Epidemiology, Truncating variants

## Abstract

Heart failure (HF) is a complex clinical syndrome defined by the inability of the heart to pump enough blood to meet the body’s metabolic demands. Major causes of HF are cardiomyopathies (diseases of the myocardium associated with mechanical and/or electrical dysfunction), among which the most common form is dilated cardiomyopathy (DCM). DCM is defined by ventricular chamber enlargement and systolic dysfunction with normal left ventricular wall thickness, which leads to progressive HF. Over 60 genes are linked to the etiology of DCM. Titin (TTN) is the largest known protein in biology, spanning half the cardiac sarcomere and, as such, is a basic structural and functional unit of striated muscles. It is essential for heart development as well as mechanical and regulatory functions of the sarcomere. Next-generation sequencing (NGS) in clinical DCM cohorts implicated truncating variants in titin (TTNtv) as major disease alleles, accounting for more than 25% of familial DCM cases, but these variants have also been identified in 2–3% of the general population, where these TTNtv blur diagnostic and clinical utility. Taking into account the published TTNtv and their association to DCM, it becomes clear that TTNtv harm the heart with position-dependent occurrence, being more harmful when present in the A-band TTN, presumably with dominant negative/gain-of-function mechanisms. However, these insights are challenged by the depiction of position-independent toxicity of TTNtv acting via haploinsufficient alleles, which are sufficient to induce cardiac pathology upon stress. In the current review, we provide an overview of TTN and discuss studies investigating various TTN mutations. We also present an overview of different mechanisms postulated or experimentally validated in the pathogenicity of TTNtv. DCM-causing genes are also discussed with respect to non-truncating mutations in the etiology of DCM. One way of understanding pathogenic variants is probably to understand the context in which they may or may not affect protein–protein interactions, changes in cell signaling, and substrate specificity. In this regard, we also provide a brief overview of TTN interactions in situ. Quantitative models in the risk assessment of TTNtv are also discussed. In summary, we highlight the importance of gene–environment interactions in the etiology of DCM and further mechanistic studies used to delineate the pathways which could be targeted in the management of DCM.

## Introduction

Heart failure (HF) is a complex clinical syndrome which concerns the impaired ability of the heart to pump and/or fill with blood, resulting in inadequate cardiac output to meet metabolic demands or, more commonly, adequate cardiac output but only due to compensatory neurohormonal activation (Mann and Bristow 2005). The prevalence of HF in the general population is high (1–1.5%) and morbidity and mortality is among the highest of any disease or disease syndromes (Ho et al. 1993; Roger 2013). HF is probably best understood from the vantage point of cardiomyopathies. Cardiomyopathies are a heterogeneous group of diseases of the myocardium associated with mechanical and/or electrical dysfunction that usually exhibit inappropriate ventricular hypertrophy or dilatation, with frequently occurring underlying genetic causes (Maron et al. 2006). Cardiomyopathies are classified into primary and secondary cardiomyopathies. Primary cardiomyopathies are based on the exclusion of secondary cardiomyopathies, and include several clinical types, of which the most common is dilated cardiomyopathy (DCM), which is characterized by ventricular chamber enlargement and systolic dysfunction with normal left ventricular wall thickness, leading to progressive HF and a decline in left ventricle (LV) contractile function, ventricular and supraventricular arrhythmias, thromboembolism, and sudden or HF-related death (Maron et al. 2006). DCM has a prevalence of up to 1 in 250 (Schafer et al. 2016) and is the third most common cause of HF, being the most frequent cause of heart transplantation. It is more common in males between the ages of 20 to 50 years, with the average 5-year survival after diagnosis being 50%, as patients often develop progressive congestive HF with life-threatening atrial and ventricular arrhythmias (Cohn et al. 1993; Eichhorn and Bristow 1996; Mann and Bristow 2005).

Familial or genetic DCM accounts for 20–30% of all cases, and further clinical evaluation identifies 30–50% of patients with DCM having relatives who are affected or likely to be affected (Herman et al. 2012). In the majority of cases, DCM shows an autosomal dominant (AD) transmission. However, autosomal recessive, X-linked recessive, and mitochondrial inheritances have also been reported (Chauveau et al. 2014). Familial DCM is genetically heterogenous; most families present with monoallelic and monogenic types of inheritance, while others present with multiallelic and multigenic genotypes (Chauveau et al. 2014). More than 60 genes have been implicated in the etiology of DCM (McNally et al. 2013), and recent genetic analysis implicated titin (TTN) as the predominant DCM-causing gene in multicohort studies (Herman et al. 2012; Walsh et al. 2016). Although the majority of TTN mutations exhibit pure cardiac manifestation, TTN mutations also exhibit purely skeletal muscle phenotypes (Granzier et al. 2005; LeWinter and Granzier 2013). In addition, mutations manifesting both cardiac and skeletal muscle phenotypes have also been reported (Chauveau et al. 2014).

## TTN interactome

**Fig. 1:**
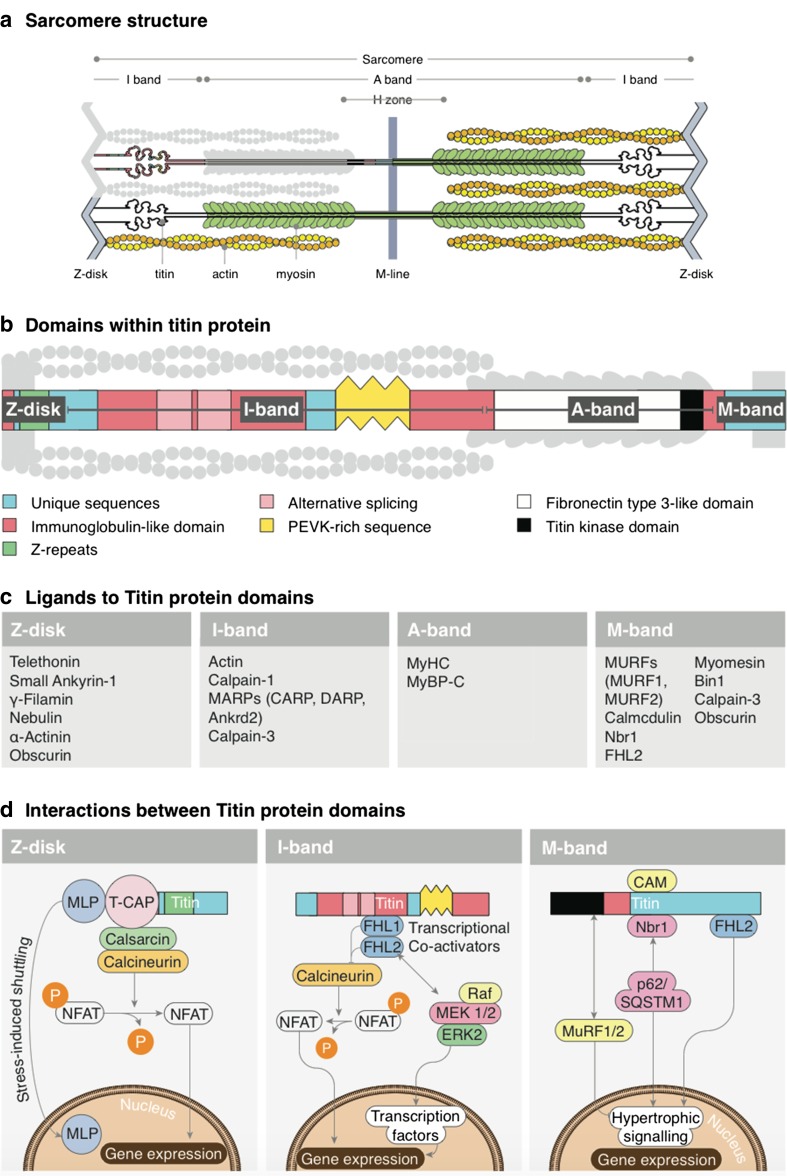
Titin (TTN), its binding partners, and signaling hotspots. **a** Simplified schematic of cardiac sarcomere with TTN. **b** Schematic diagram of TTN depicting its domains. **c** Short list of ligands interacting directly or indirectly with TTN. **d** Signaling hotspots of Z-disk TTN, I-band TTN, and M-band TTN

To understand the effects of different TTN mutations, it is important to provide a succinct overview of TTN interaction with other proteins in the sarcomere (Fig. 1). In order to obtain a comprehensive insight into TTN ligands, its signaling, and its disease relevance, we refer to other reviews (Gigli et al. 2016; Kontrogianni-Konstantopoulos et al. 2009; Kötter et al. 2014; Linke and Hamdani 2014). Regional TTN structure and its interactions are enormously varied and complex. However, with a succinct overview, readers can quickly grasp some aspects of the functional importance of truncating variants in titin (TTNtv), especially how TTNtv in different regions and bands of TTN could affect the sarcomeric signaling and stability.

## Z-disk TTN

Z-disk TTN contains the first 826 amino acids which span this structure horizontally. TTN also overlaps with the N-terminal residues of neighboring TTN molecules in an antiparallel manner, which generates N-terminal to N-terminal connections between two TTN molecules of opposite sarcomeres. Z-disk TTN is arranged into seven immunoglobulin (Ig) domains, interspersed with Z-insertion sequences. Z-disk TTN ligands are shown in Fig. 1c, d, which indicates its importance for a Z-disk-related signaling hotspot. Z-disk TTN structurally and functionally interacts with myofibrillar and sarcolemmal proteins and is, thus, important for myofibrillar assembly, stability, and signaling. TTN is also important for the extracellular milieu by anchoring via intermedia filaments to costamers. Of particular note is the Z-disk anchorage of TTN-Tcap (telethonin), considered to be essential for the functioning of the Z-disk, including its mechanosensory actions, by recruiting other interacting and signaling partners to the Z-disk (Knöll et al. 2002, 2010, 2011). Z-disk TTN also interacts with small ankyrin proteins, which further interact with spectrin, desmin, and obscurin, connecting Z-disk TTN to other cytoskeletal structures. Filamin C connects Z-disk TTN to costamers via integrin and sarcoglycans, and takes part in the Z-disk stretch-sensing pathways. Moreover, Z-disk TTN interacts with nebulin, which supports the stabilization of the Z-disk anchorage by interacting with actin, desmin, CapZ, and myopalladin. Z-disk TTN interaction with α-actinin provides additional mechanical stability (Clark et al. 2002; Gigli et al. 2016; Granzier and Labeit 2005; Kontrogianni-Konstantopoulos et al. 2009; Linke 2008; Miller et al. 2003).

## I-band TTN

I-band TTN is a highly interactive structure with great potential for alternative splicing. This results in isoforms contributing to cardiac and skeletal muscle specific phenotypes. The meta-transcript (containing principle cardiac and skeletal isoforms) of I-band TTN is mainly composed of Ig domains, cardiac N2B region, and skeletal N2A region (which contains non-repetitive sequences and Ig domains), followed by the Pro-Glu-Val-Lys (PEVK) TTN domain, all of which contribute to TTN-related elasticity (Kontrogianni-Konstantopoulos et al. 2009; Trombitás et al. 1998).

Proximal I-band TTN mainly supports sarcomeric integrity, while medial/distal I-band TTN functions as a bidirectional molecular ruler which determines the resting and passive force tension upon stretch, primarily as a function of the above-mentioned domains (Helmes and Granzier 1996). TTN also limits cardiac sarcomeric length when end-diastolic volumes increase under physiological conditions, and, thus, plays a role in determining length-dependent activation of cardiac muscles, which forms the basis of the Frank–Starling mechanism (Cazorla et al. 2001; Fukuda and Granzier 2005). It also controls the displacement range when stretched beyond the upper limit of sarcomeric length during ventricular diastole (Kontrogianni-Konstantopoulos et al. 2009).

I-band TTN also works as a biochemical stress sensor via its interactions with αβ-crystalline, DRAL, FHL1, and FHL2 via the Gαq-MAPK pathway, and may regulate muscle gene expression via its interaction with MARPs family members, which shuttle to the nucleus and which may interact with various transcription factors (Granzier and Labeit 2005; Hojayev et al. 2012).

Finally, I-band TTN is involved in a sarcomeric quality control pathway via its interactions with Ca^+2^-dependent protease Calpain-1, Calpain-3, and also acts as a reservoir of inactive Calpain-3 (Granzier and Labeit 2004; Witt et al. 2004).

## A-band TTN

A-band TTN mainly consists of Ig and fibronectin (FN-III) motifs. FN-III motifs are exclusive to A-band TTN. Ig and FN-III are further arranged into two super-repeats, in which FN-III domains are bisected by Ig domains. In contrast to I-band TTN, A-band TTN is inextensible, as it provides binding sites for myosin, and, hence, functions as a stable anchor. TTN binding to myosin S1 fragments also plays a role in regulating Ca^+2^ sensitivity (Muhle-Goll et al. 2001). Super-repeat A-band TTN domains interact with and help localizing sarcomeric MyBP-C. A-band TTN has interaction sites for muscle ring finger proteins (MURF1 and MURF2), in which MURF1 is suggested to play an important role in quality control and protein turnover pathways in the center of the sarcomere, while the interaction of MURF2 with A-band TTN plays a role in the formation of mature A-band structures in the sarcomere (Fig. 1) (Granzier and Labeit 2005; Kontrogianni-Konstantopoulos et al. 2009). Mutations in the A-band TTN are implicated as predominant genetic causes of DCM (Akinrinade et al. 2016; Schafer et al. 2016; Yoskovitz et al. 2012) .

## M-band TTN

M-band TTN contains a putative serine/threonine kinase domain as its prominent feature and, also, Ig-CII domains interspersed with M-insertion sequences. The functional role of M-band TTN largely revolves around its kinase domain activities, although the kinase activity has recently been challenged (Bogomolovas et al. 2014). It is probably involved in stress-sensing mechanisms via Ca^+2^-calmodulin regulated mechanochemical signal transduction pathways (Gautel et al. 1995; Kontrogianni-Konstantopoulos et al. 2009). M-band TTN is implicated in sarcomerogenesis by making a scaffold with myomesin, which, in turn, links M-band TTN to myosin thick filaments (Musa et al. 2006). This myomesin–TTN–myosin axis is important for M-band stability. In addition, it is also a metabolic stress sensor via its interacting ligands DRAL/FHL-2, which tethers metabolic enzymes to M-band TTN, and is involved in ubiquitin-mediated turnover via its interaction with a zinc finger protein nbr1, p62, MURF1, and MURF2 (Kötter et al. 2014). M-band TTN interacts with MURF2, which is thought to play a role in cardiac development (McElhinny et al. 2004). Probably another important interaction takes place at the extreme COOH-terminal end of M-band TTN, where an TTN/Calpain-3/P94 interaction occurs and which plays a role in the turnover of M-band-associated proteins (Beckmann and Spencer 2008; Granzier and Labeit 2005). A TTN kinase mutation (R279W) has initially been described as causative for hereditary myopathy with early respiratory failure (HMERF) (Lange et al. 2005). However, the discovery of another HMERF case, where this mutation has clearly been excluded as the disease-causing event (Tasca et al. 2010), in addition to other cases where mutations in TTN fibronectin domains (especially the 119th fibronectin-3 domain) have been identified (Pfeffer et al. 2012, 2014), render a disease-causing role for this mutation unlikely. Mutations of M-line TTN are shown to cause recessive titinopathy (TTN-induced pathology) manifesting itself in the cardiac as well as skeletal involvement (Carmignac et al. 2007; Yoskovitz et al. 2012).

## TTN signaling hotspots

A complex network of endocrine, neuroendocrine, paracrine, and autocrine signaling modulates the activity of TTN in the pathophysiology of cardiomyopathy. A comprehensive overview of these signaling activities is beyond this review however, with at least three signaling hotspots being worthy of mention and which exist along the length of TTN: in Z-disk TTN, in A-band TTN, and in M-band TTN.

The TTN-T-cap-MLP axis appears to be an important stretch-sensing complex which is localized to the Z-disk (Knöll and Buyandelger 2011; Knöll et al. 2002; Kontrogianni-Konstantopoulos et al. 2009) and which provides direct links to hypertrophic/atrophic signaling via its interaction with calsarcin and, thus, is involved in the activation of the calcineurin/nuclear factor of activated T cells (NFAT) pathway. Calcineurin is a calcium/calmodulin-dependent serine/threonine phosphatase which dephosphorylates NFAT to allow its nuclear translocation and activation of pro-hypertrophic genes (Heineke and Molkentin 2006; Olson and Williams 2000).

Another important biochemical stress sensor identified in cardiac I-band TTN functions via N2-B/FHL-1/MAPK signaling, where FHL1 and FHL2 proteins have their binding sites (Sheikh et al. 2008; Lange et al. 2005). FHL1 may signal through mitogen-activated protein kinase (MAPK) signaling (which includes MEK1/2, their activator Raf-1, and ERK2) (Sheikh et al. 2008). Upon stretch, MEK1/2-induced phosphorylation of ERK1/2 leads to its translocation into the nucleus and the activation of target transcription factors such as c-Myc, c-Fos, and cAMP response element-binding (Mebratu and Tesfaigzi 2009). FHL2 is also involved in I-band TTN signaling and possibly represses the calcineurin/NFAT pathway (Hojayev et al. 2012; Kontrogianni-Konstantopoulos et al. 2009; LeWinter and Granzier 2014).

Another signaling axis is probably formed by the putative TTN kinase domain via (TK)-Nbr1-p62 and MURFs in the M-band region. MURFs work in concert with other transcription factors in order to suppress the hypertrophic gene expression program, whereas the TK-Nbr1-p62 axis activates pro-hypertrophic gene expression (Granzier and Labeit 2005; Kontrogianni-Konstantopoulos et al. 2009; LeWinter and Granzier 2014; Linke 2008).

## TTN isoforms

TTN is a unique protein with highly extensible (I-band TTN) and inextensible regions (Z-disk TTN, A-band TTN, M-band TTN). In addition, it contains regions undergoing extensive regional alternative splicing (in particular, A-band TTN) and regions which are constitutive (Z-disk TTN, I-band TTN, M-band TTN). Alternative splicing in the A-band TTN results in the cardiac-specific N2B isoform with interspersed unique sequence (N2B-Us), N2BA, which contains the N2B and N2A sequence, and skeletal isoform N2A (without N2B sequence). All these isoforms contain the extensible PEVK region. Cardiac isoforms contain three extensible regions: tandem Ig segments, PEVK segments, and cardiac-specific N2B-Us. Upon increasing their length, Ig segments stretch first, followed by PEVK and, finally, N2B-Us segments, which stretch at the upper limit of physiological sarcomeric length (Guo et al. 2010; Trombitás et al. 1999). Whether TTN leads the passive tension in cardiac sarcomeres is determined by the expression ratio of N2BA and N2B isoforms and by TTN phosphorylation (Kötter et al. 2014). During development, the N2BA/N2B ratio decreases in parallel with a TTN-related loss of elasticity (Lahmers et al. 2004), whereas in HF conditions, the N2BA/N2B ratio increases, which, in turn, increases in TTN compliance (Kontrogianni-Konstantopoulos et al. 2009; LeWinter and Granzier 2014). The overall higher expression of N2B is observed in healthy hearts and N2BA is observed in hearts with systolic dysfunctions (DCM). Functional alterations due to mutation in the N2B region are reported in DCM as well as in the hypertrophic cardiomyopathy (HCM) phenotype (Matsumoto et al. 2005). Endocrine regulation by triiodothyronine (T3), insulin, and angiotensin II seems to play an important role in TTN isoform switching. Consistent with this, in HF biopsies and in animal models of HF, differential potency of endocrine regulators has been shown to vary the N2BA/N2B ratio in failing hearts (Krüger et al. 2008, 2010). TTN isoform switching is further regulated by the splicing factor RNA-binding motif protein-20 (RBM20). A lack of RBM20 causes the expression of aberrant N2BA isoforms in failing hearts (Guo et al. 2012). RBM20 is expressed preferentially in cardiac tissue and implicated in cardiomyopathies, while RBM20 antagonists represent a potential therapeutic target in HF management (Bull et al. 2016; Li et al. 2010; Methawasin et al. 2014, 2016; Zhu et al. 2015).

## TTN mutations

TTN is a DCM locus (Siu et al. 1999), localizes to chromosome 2q31, and is encoded by 363 exons from which a 100-kb mRNA is transcribed. With up to 4200 kDa and up to 38,000 amino acids, TTN is the largest protein known in biology and resides within cardiac and skeletal sarcomeres (Kontrogianni-Konstantopoulos et al. 2009). TTN has huge potential of being alternatively spliced, leading to different protein isoforms. Increasing complexity in protein isoforms is also brought by truncating variants which can be introduced into canonical sequences, as a consequence of various mutations which lead to premature termination codons (PTC), e.g., point mutations (nonsense mutation) or insertions/deletions inducing frameshifts and mutations disrupting the canonical splice sites of exons, resulting in out-of-frame transcripts with premature truncations. Mechanisms have been proposed (or experimentally observed otherwise) to demonstrate how TTNtv lead to cardiac phenotypes. A straightforward explanation is that, once the truncated TTN gets incorporated into the sarcomere, it is unable to function normally, thus leading to gain-of-function/dominant negative phenotypes (Roberts et al. 2015). Contrary to the dominant negative mechanism, the majority of truncated transcripts are degraded by the cell via nonsense-mediated mRNA decay (NMD), which is thought to rescue dominant negative effects of truncated proteins, but leaves the cells in a haploinsufficient state which can also lead to the disease phenotype (Zhou et al. 2015). Dominant negative alleles might be more pathological than simply lacking functional TTN protein, because it can alter the substrate binding/activity of TTN ligands or even bind to spurious ligands, activating pathogenic signaling. On the other hand, the haploinsufficient state in heterozygotes can lead to suboptimal responses and compensatory changes in the ventricular wall prototypic of DCM in response to increased cardiac stress. However, despite considerable research being done, the exact mechanism of TTNtv-induced DCM is not clear (i.e., do all TTNtv act via dominant negative or haploinsufficient or both mechanisms? Are competition effects at play? And/or are other mechanisms at play? Does biomechanical stress play a role or does stress exacerbate the disease?).

## TTNtv in DCM

Prior to next-generation sequencing (NGS), routine analysis of the TTN gene had been extremely challenging because of its size and complexity. Thus, only a few TTN mutations had previously been reported, and the general incidence and spectrum of titinopathies was significantly underestimated. Early work pointed towards the importance of TTN mutations in HCM (a myocardial disease defined by a hypertrophied, non-dilated LV in the absence of another systemic or cardiac disease; Maron et al. 2006) (Bos and Ackerman 2010; Satoh et al. 1999; Wang et al. 2010a). They identified TTN mutations localized to the Z-disk domain, and affecting passive elasticity, thus linking these mutations to mechanosensation (Bos et al. 2006; Satoh et al. 1999). In addition, TTNtv were identified in zebrafish and in families affected by DCM (with a segregating 2 base pair insertion in TTN and 1 base pair deletion mutation in TTN) (Gerull et al. 2002, 2006; Itoh-Satoh et al. 2002; Xu et al. 2002). These early reports provided powerful evidence of a disease-causing role of TTN mutations in the pathogenesis of DCM in experimental HF models and in patients, respectively.

The existence of very large size, intricate modular organization, alternatively spliced isoforms, and newly identified internal promoter (Cronos) in TTN, are all factors that have generated considerable challenges in demystifying the functional effects of TTNtv in disease penetrance, allele dosage, and by posing challenges to clinicians in genetic risk assessment for cases identified positive for TTNtv with or without overt cardiac phenotypes (Chauveau et al. 2014; Zou et al. 2015). The involvement of TTN mutations, and especially of TTNtv in the pathogenesis of cardiomyopathies, have been known for more than a decade, but the lack of sophisticated sequencing techniques has not allowed the identification and population prevalence of TTNtv in large cohorts. With technical advancements in sequencing technology, genome-wide association studies (GWAS) have implicated hundreds of loci in cardiovascular diseases (CVD). Nevertheless, the progression of GWAS in relation to clinical management and therapeutics has not kept pace, owing largely to the inability to separate causal genes from bystanders (Haas et al. 2015; Wain 2014).

**Fig. 2:**
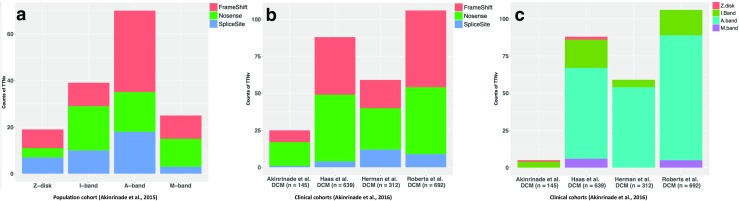
Distribution of titin-truncating variants (TTNtv) in population (Akinrinade et al. 2015a) (**a**) and dilated cardiomyopathy (DCM) cohorts (**b**, **c**) evaluated by Akinrinade et al. (2016)

Clinical assessments have identified 30–50% of all DCM patients as having relatives who are affected or likely to be affected (Chauveau et al. 2014; Herman et al. 2012). With advancements in NGS technologies, genetic epidemiology studies have shown that TTNtv accounts for about 25% of these familial DCM cases, but the interpretation of such TTNtv has been obscured because TTNtv have also been identified in 2–3% of the general population, where carriers do not exhibit overt HF signs or symptoms (Herman et al. 2012). Our ability to evaluate the pathogenicity of TTNtv largely relies on the variants in the signal-to-noise ratio (case-to-control), segregation studies within families, and the use of animal/cellular models to predict pathogenicity. In order to improve the clinical and diagnostic potential of TTNtv, recent studies employing NGS have been conducted with clinically well characterized DCM cohorts and compared them with population cohorts (comprising individuals without overt cardiac phenotypes) in order to delineate the frequency of TTNtv and their functional relevance. DCM cohort data are compared with genetic sequencing data from population cohorts which are available publicly [1000 Genomes Project, Exome Sequencing Project (ESP), and Exome Aggregation Consortium (ExAC)] (Akinrinade et al. 2015a; Bahcall 2016). In exploiting these public datasets, Akinrinade et al. (2015a) comprehensively analyzed these datasets in order to capture the population distribution of TTNtv. An immediate observation from their study was the low prevalence of TTNtv in the general population, with increasing technical advancements in sequencing chemistries and in bioinformatics pipelines being used to confidently assign the true population distribution of TTNtv. Almost half of the identified TTNtv were located in exons with low percentages spliced in (PSI: fraction of mRNAs that represent the inclusion isoform). These TTNtv were referred to as alleles with a low probability of pathogenicity. Pathogenic or likely pathogenic TTNtv (TTNtv which affect all transcripts; transcripts having high PSI) appear to have a frequency of about 0.35% in the general population (averaged over the 1000 Genomes Project, ESP, and ExAC) when compared to 2–3% TTNtv in population cohorts, as has been observed previously (Herman et al. 2012). Population distribution of nonsense mutations were most prevalent, followed by frameshift and splice site mutations, which also result in TTN truncation (Fig. 2a) (Akinrinade et al. 2015a).

In this review, we will discuss four recent comprehensive studies which analyze TTNtv in their respective clinical DCM cohorts (i.e., Herman et al. 2012, Haas et al. 2015, Roberts et al. 2015, and Akinrinade et al. 2015b). These DCM cohorts were further analyzed and pooled to obtain a better idea of the overall relevance of TTNtv in DCM by Akinrinade et al. (2016). Further, a recent study from a Swedish DCM cohort (*n* = 176) also described TTNtv high enrichment in DCM compared to the control 1000 Genomes cohort (Dalin et al. 2017). Overall, the total DCM truncating mutations reported in these studies (from the studies of Herman et al. 2012, Hass et al. 2015, Roberts et al. 2015, Walsh et al. 2016, and Dalin et al. 2017) are 308 (nonsense mutations = 127, frameshift mutations = 127, splice site mutations = 54, indel mutation = 1). Further, 36 TTNtv (nonsense mutations = 7, frameshift mutations = 23, and splice site mutations = 6) are described in the literature (Chauveau et al. 2014). Akinrinade et al. (2015b) and Roberts et al. (2015) described 47 TTN-truncating mutations (nonsense mutations = 16, frameshift mutations = 25, splice site mutations = 6) in apparently healthy individuals without overt DCM phenotype. A further detailed list of all TTN mutations published to date can be located in the public databases from OMIM (http://omim.org/entry/188840), HGMD (http://www.hgmd.cf.ac.uk/ac/index.php), and/or LOVD (http://www.LOVD.nl/TTN). We will discuss individual cohorts in their respective studies, and we will then present the re-evaluation of the pooled cohorts analyzed by Akinrinade et al. (2016). In the studies by Herman et al. (2012) and Roberts et al. (2015), they included non-essential splice sites mutations and missense mutations in exons with low PSI in their estimation of TTNtv in the DCM cohorts. This results in a relatively high prevalence of TTNtv in DCM. The missense variants might be non-pathogenic because of their location in TTN exons with low PSI and the high background frequencies in population cohorts, which highlights the need for further studies in order to evaluate the importance of missense mutations in the pathogenesis of HF. Overall, Herman et al. (2012) reported 72 mutations (25 nonsense, 23 frameshifts, 23 splicing, and one large tandem insertion), which were over-represented in the TTN A-band that altered full-length TTN, and labeled these TTNtv with high preponderance of being DCM alleles with high penetrance in the elderly, as typically observed for alleles with dominant inheritance. Cardiac outcome was similar between cases with and without TTNtv, which highlighted a similar course of progression in the later stages of disease (e.g., fetal activation of gene expression) of DCM with and without TTNtv. In the study performed by Haas et al. (2015), TTN stood out as the major disease-causing gene, with high rates of mutation compared to the total number of mutations in the panel of disease-causing genes selected in their study (Haas et al. 2015). Overall, they reported 39 nonsense, 34 frameshift, and four splice site mutations in their DCM cohort. However, no clear preponderance of TTNtv in the A-band was reported in their study.

A recent study by Roberts et al. (2015) sequenced TTN in a discovery cohort, which included a population cohort from the Framingham Heart Study and Jackson Heart Study (FHS and JHS, respectively), and clinical cohorts of prospectively enrolled unselected ambulatory DCM patients and end-stage DCM patients. Again, TTNtv were reported to be enriched in A-band TTN in unselected and end-stage DCM cohorts when compared to the population cohort. Consistent with previous findings, TTNtv in clinical cohorts affected constitutive exons (exons with high PSI) compared to the population cohorts. TTNtv targeting both N2B and N2BA isoforms were more strongly associated (odds ratio: 19) with DCM compared to TTNtv observed only in the N2BA isoform (odds ratio: 3.8). A negative correlation of TTNtv with alternative splicing was reported with the higher occurrence of TTNtv in exons with lower alternative splicing (i.e., high PSI) in end-stage DCM subjects when compared to unselected DCM, and vice versa in healthy volunteers and population cohorts. These findings strongly corroborate the fact that TTNtv-affected exons are constitutive and, hence, are more likely to be disease-causing. TTNtv identified by Roberts et al. (2015) reported >93% likelihood of being pathogenic when identified in unselected DCM cases and an even higher likelihood of being pathogenic when identified in end-stage DCM cases. They concluded that the pathogenic potential of TTNtv is principally determined by the exon usage and variant location, and postulated that these two factors could help identify pathogenic variants from those that are benign.

The high prevalence of TTNtv in a Finnish cohort of DCM patients has also been reported by Akinrinade et al. (2015b). Among the variants identified in this cohort, TTNtv (nonsense, frameshift) were highly enriched (53%) when present in constitutive exons. When compared to the Herman et al. (2012) report, where TTNtv were listed as 25% in familial and 18% in sporadic DCM cases, the Akinrinade et al. (2015b) report tabled more conservative estimates, with 20.6% in familial and 14.6% in sporadic Finnish DCM cases. They also reported a very low frequency of DCM cases with compound mutations in DCM genes, a finding more consistent with the autosomal dominant inheritance of familial DCM. TTNtv were also identified as major genetic contributors towards DCM (22 TTNtv in DCM compared to none in the control population) in the Swedish DCM cohort analyzed by Dalin et al. (2017). Their study was limited in terms of TTN gene coverage for sequencing; however, it concluded with findings that TTNtv in DCM are associated with reduced survival. TTNtv are also recently reported in anthracyclines-treated breast cancer patients who developed DCM within months after receiving the chemotherapy (Linschoten et al. 2017). Further, TTNtv in the etiology of DCM is also confirmed by sequencing TTN in peripartum cardiomyopathy, which is a pregnancy-induced cardiomyopathy with clinical characteristics similar to DCM (van Spaendonck-Zwarts et al. 2014). Ware et al. (2016) reported that 15% of identified TTNtv in peripartum cardiomyopathy shared TTNtv with DCM, and proposed shared mechanisms responsible for both types of cardiomyopathies. All these observations point towards the pathogenicity of TTNTv and the high risk of developing DCM.

Taking into account the heterogenicity in the bioinformatics pipelines in individual studies, Akinrinade et al. (2016) further pooled the individual DCM cohorts (published in the studies discussed above), which resulted in a single DCM cohort of 1788 patients and which re-evaluated the relevance of TTNtv with respect to a stringent variant-calling pipeline, filtering the TTNtv by quality score, sequencing depth, and the exclusion of rare cardiac isoforms, excluding TTNtv which affected exons with low PSI values and filtering the TTNtv overlapping in the population cohorts (ExAC) (Akinrinade et al. 2016). They concluded that TTNtv affecting all transcripts (exons with high PSI) are more prevalent in clinical cohorts (i.e., in pooled and in individual DCM cohorts), and that these variants have at least a 97% risk of being disease alleles, even when identified in unselected DCM cases. The distribution of these high-risk alleles was reported to concentrate in the A-band and as well as in the I/A band junction of TTN. A high occurrence of TTNtv in the A-band is considered clinically relevant for the DCM phenotype (Fig. 2b, c). It is possible that the A-band TTNtv escape nonsense-mediated decay, which results in the incorporation of truncated TTN into the sarcomere, thereby disturbing various functions, signaling, and protein–protein interactions, and inducing the DCM phenotype via dominant negative mechanisms. Within the A-band, the highest occurrence of TTNtv was reported in the distal A-band, which might corroborate the dominant negative mode of action for TTNtv with high disease penetrance. Vice versa, the absence of distal A-band TTNtv in population cohorts points towards their pathogenicity and incompatibility with the sustaining of life. In support of this, Gramlich et al. (2009) introduced a TTNtv (frameshift) in the distal A-band (exon 326) into the mouse genome, and reported heterozygous mutations recapitulating the human DCM phenotype upon application of biomechanical stress, while homozygous mice die in utero, thereby confirming high lethality conferred by distal A-band TTNtv.

In summary, Akinrinade et al. (2016) reported high incidences of TTNtv in the A-band (12.3%; odds ratio: 70.4) in clinical cohorts compared to 0.19% in the population cohort, which provides further support for the pathogenicity of TTNtv in the A-band regions (Fig. 2b, c). However, the occurrence of TTNtv in A-band TTN in population cohorts requires further explanation, perhaps acting as allelic factors exhibiting subclinical phenotypes upon relevant exposure. TTNtv represent high-risk alleles, particularly when present in A-band TTN and when affecting constitutive exons. However, their study did not seem to exclude cases overlapping in cohorts included in their estimation, and, thus, might have slightly overestimated the overall occurrence and distribution of TTNtv in DCM cases. These cohorts were further re-evaluated by Deo (2016) in order to build qualitative models to explain the distribution of TTNtv in the pathogenesis of DCM (discussed later).

Overall, these studies highlight the association of TTNtv with DCM and, especially, the high risk conferred by TTNtv when they reside in the A-band. Knowledge of mutational hotspots in the A-band in DCM patients could be exploited for the development of genotype-guided risk assessment in DCM management and for the development of genotype-lead therapies.

In an effort to delineate the functional impact of TTNtv, Schafer et al. (2016) performed a comprehensive analysis of TTNtv by generating rat models harboring TTNtv at Z-disk TTN (TTNtvZ) and TTNtv at the A-band TTN (TTNtvA). While TTNtvZ and TTNtvA alleles led to the synthesis of truncated TTN isoforms from both loci, the NMD of these truncated variants did not lead to the identification of dominant negative isoforms in the hearts of mutant rats, which highlighted the fact that TTNtv-induced truncated TTN follows position-independent NMD pathways in the emergence of the HF phenotype. However, is this also the case for a broader population of TTNtv in human DCM cohorts? This question will require further experimental elaboration and should be further explored in these models by employing NMD inhibitors to understand the role of dominant negative proteins if they become incorporated into the sarcomere of mutant hearts after the experimental models are treated with NMD inhibitors. Nevertheless, NMD intervention could open doors to novel therapeutics in the management of DCM. Similar to the NMD outcome for TTNtvZ and TTNtvA alleles, gene expression and metabolic signatures were also similar between TTNtv genotypes, which raised further questions with regard to the paradigm of position-dependent effects of TTNtv and HF signaling (briefly discussed later). Using advanced cardiac magnetic resonance imaging techniques, genotype positive–phenotype-negative asymptomatic individuals were reported to harbor eccentric remodeling of the myocardium, which can adversely affect the individuals in future. The observation that hearts harboring TTNtv are already alerted with metabolic stress signaling, and that a further increase in metabolic stress might not be compensatable, is consistent with the age-induced onset of DCM in TTNtv-positive individuals (Schafer et al. 2016).

One of the questions arising naturally from these observations is whether the TTNtv are sole etiological factors in DCM or whether they act as a risk factor in DCM. Functional studies derived from mice, rat, and zebrafish models show that heterozygous animals are apparently healthy with normal or near to normal cardiac functions, without signs and symptoms of DCM or HF. This leads us to believe that contributions from etiological factors other than TTNtv precipitate towards a DCM phenotype in the background of genetic stress (i.e., TTNtv), and that the required additional stress for the DCM to emerge means that TTN insufficiency alone is unlikely to be the sole cause of DCM. Another conclusion drawn from TTNtv epidemiology studies is the differential sensitivity of TTN mutations along its length towards the DCM phenotype. It appears that I-band TTNtv are less prevalent and perhaps more tolerable compared to A-band TTNtv. As has been stated earlier in the TTN signaling section, I-band TTN is mainly a substrate for signaling and metabolic activity as compared to A-band TTN, which provides structural and functional interactions with major sarcomeric contractile protein, such as myosin heavy chain and myosin binding protein C. With A-band TTNtv, such interactions are severely compromised in DCM patients and/or are at risk from defective functions relating to stress in genotype-positive asymptomatic individuals. I-band haploinsufficiency or I-band dominant negative TTN might still contribute towards signaling events occurring at I-band TTN on rest or stress conditions without severely impairing the cardiac conditions. Further, A-band TTN exons were constituted by high PSI values compared to I-band TTN, indicating that any mutation in A-band will affect most or all of the splice variants and, hence, be more deleterious, whereas mutations in I-band variants could spare splice variants and, hence, have milder outcome. The Schafer et al. (2016) study also pointed towards the importance of TTNtv lethality in exons with high PSI, but they reflected this effect regardless of which TTN region is being affected, rather than other studies which pointed out the importance of TTNtv in high PSI exons in the A-band region. These observations highlight the functional aspects of TTNtv, but the question still remains open as to which factors make A-band TTN more susceptible to truncating mutations, e.g., whether the deamination of CpG dinucleotides or the topological organization of this region within the 3D genome architecture remains largely unclear.

Taking into consideration the findings from large multicohort studies delineating TTNtv pathogenicity in DCM and identification of an internal TTN promoter named Cronos which could rescue the N-terminal TTN truncation (Zou et al. 2015), Deo (2016) proposed a quantitative classification model for clinical use in order to classify and predict the pathogenicity of TTNtv in clinical and population cohorts. This model incorporated three factors accounting for the distribution of TTNtv: (1) alternative splicing, (2) disruption of internal promoter Cronos, and (3) whether the distal C-terminus was targeted. This model predicted a steady increase in the risk of DCM with TTNtv in exons with increasing PSI, recapitulating the previous findings that TTNtv are more prevalent in exons with high PSI values. The risk of TTNtv disrupting the Cronos was also high in DCM cohorts relative to the general population. An interesting observation that is not obvious from previous studies was a predicted low pathogenicity of TTNtv occurring at the distal C-terminus end of TTN, partially attributable to the preserved functionality of the distal C-terminus truncated proteins. Although such models could be instrumental in stratifying the disease risk carried by genetic variants, a multifactorial disorder such as DCM probably requires much more comprehensive models to be able to address gene–environment interactions in stratifying the differential risk carried by TTNtv in response to environmental triggers, i.e., exposure to an environmental stressor [e.g., body mass index (BMI), older age, diet, diabetes, or other evolving comorbidities], which, finally, may precipitate the DCM phenotype. Recently, in view of these developments, the World Heart Federation (http://www.world-heart-federation.org) published a new classification scheme for cardiomyopathies, called MOGE(S) (morphofunctional, organ involvement, genetic or familial, etiology, stage), which accounts for the environmental triggers in the quantitative model in the background of genetic susceptibility to DCM. Implementation of MOGE(S) classification in a pilot study improved the stratification of patients with DCM, most likely by using the combination of genetic evaluation and non-genetic, environmental factors (Hazebroek et al. 2015). Environmental triggers provide the causes of epigenetic changes in cardiovascular pathophysiology (Handy et al. 2011) and also, in the context of TTNtv-induced DCM phenotypes, complex epigenetic regulations might exist at multiple levels, namely, the emergence of genotype-specific epigenetic signatures, together with the environment shaping the TTNtv epigenome (genotype–epigenotype–environment interaction), in the etiology of DCM and related cardiomyopathies. Preliminary studies have shed some insight into the altered DNA methylation changes in human DCM subjects (Haas et al. 2013; Jo et al. 2016). DCM patients are rendered more complex, with an overlapping spectrum of phenotypic disorders, which confounds the true distribution of DCM-induced epigenetic changes. These inconsistencies need to be addressed in well-characterized DCM models in the future.

## Animal models of TTN mutations

**Fig. 3:**
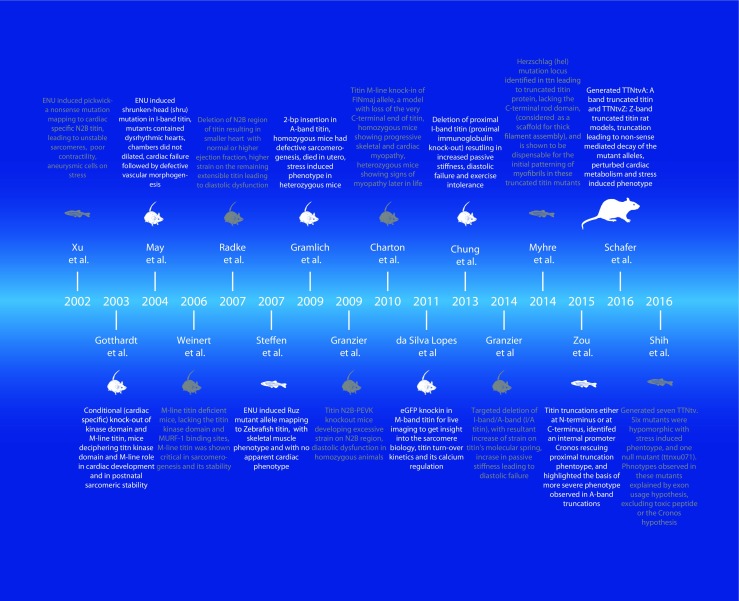
A timeline representation of animal models generated to study TTN mutations. *ENU* Ethylnitrosourea; *PEVK* Pro-Glu-Val-Lys TTN domain; *eGFP* enhanced green fluorescent protein

TTN is a giant protein with a complex modular structure, tissue-specific isoform regulation, which interacts with >25 other proteins and many signaling pathways converge into TTN’s different domains. The aim of animal models was to simplify this complexity of TTN into manageable research questions. Although a number of small animal models could be employed, such as rat, mice, and zebrafish, which are preferable for use in TTN research, the choice largely depends on the convenience, pathophysiological relevance of the host, and cost involved. Traditionally, mouse models have been instrumental in delineating the pathophysiology of M-band TTN (Charton et al. 2010; Gotthardt et al. 2003; Weinert et al. 2006), I-band TTN sarcomere (Chung et al. 2013; May et al. 2004), N2B and N2B-PEVK TTN (Granzier et al. 2009; Radke et al. 2007), A-band TTN (Gramlich et al. 2009), A/I-band TTN (Granzier et al. 2014), and visualizing sarcomeric kinetic and turnover (da Silva Lopes et al. 2011). Rat models are used to study the pathophysiology of TTNtv at Z-disk or A-band regions of TTN (Schafer et al. 2016). Recently, zebrafish models emerged as powerful model organisms to study hypertrophy and HF. Many research groups addressed knowledge gaps in titinopathies using zebrafish models and validated large-scale genetic screens (such as Crispr/Cas9) (D’Agostino et al. 2016; Varshney et al. 2015) and performed functional assays (echocardiography) (Hein et al. 2015). Zebrafishes have also been used in cardiac reverse phenotyping of ethylnitrosourea (ENU)-induced chemical mutagenesis (Myhre et al. 2014; Steffen et al. 2007; Xu et al. 2002) and introducing TTNtv in the zebrafish genome (Shih et al. 2016; Zou et al. 2015). In Fig. 3, we provide a pictorial timeline representing animal models generated to study TTN mutations, with emphasis on TTNtv.

**Fig. 4:**
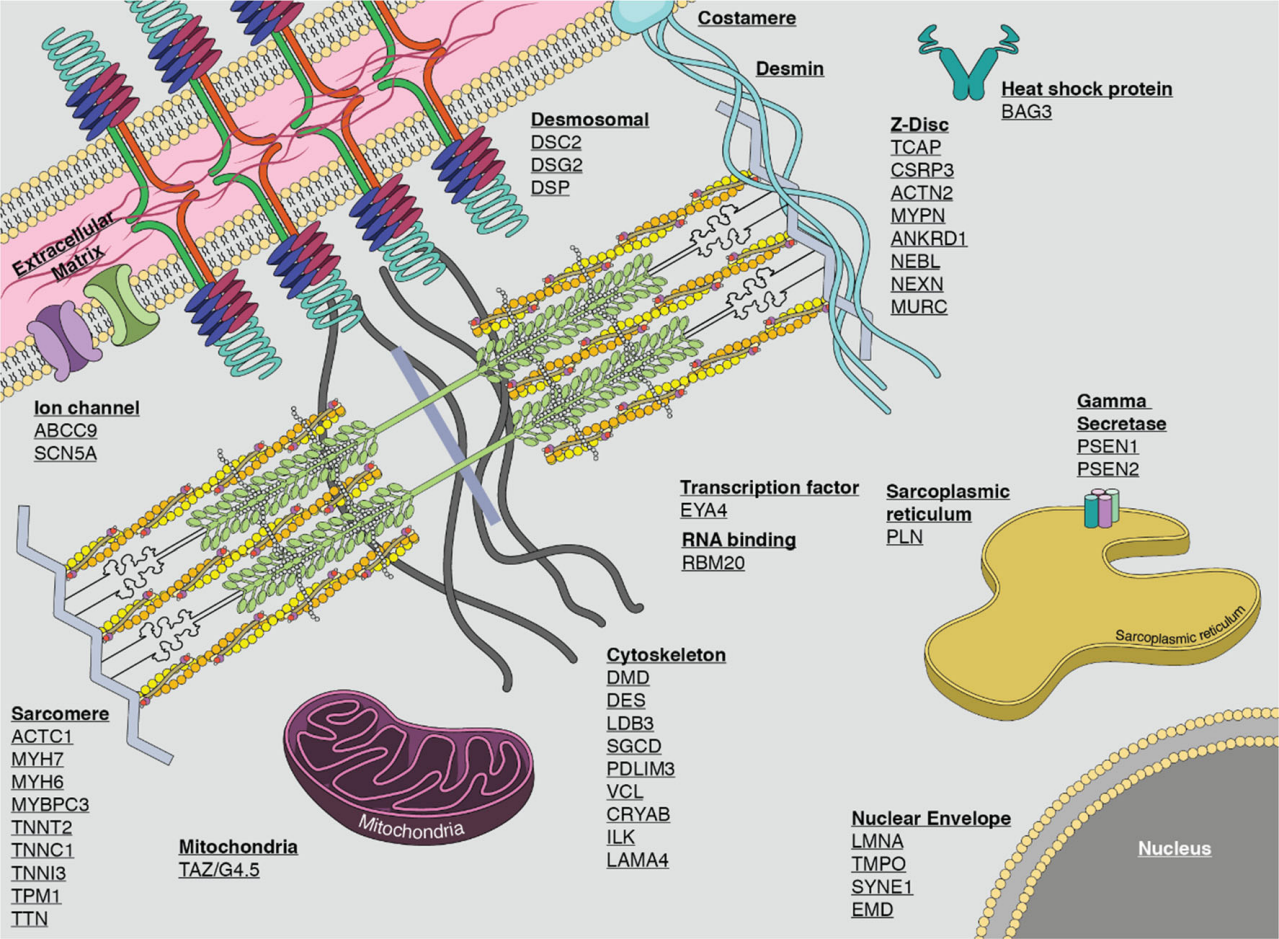
Schematic section of cardiomyocyte depicting genes associated with non-syndromic familial DCM (Hershberger et al. 2013). Sarcomeric gene TTN is the major DCM gene, accounting for 25% of familial DCM cases

In this context, it is probably important to mention that rare variants in TTN are not the only causal alleles in the genetic etiology of DCM. In fact, rare variants (both truncating and non-truncating) have also been described in sarcomeric genes, Z-disk genes, cytoskeletal genes, ion channels genes, nuclear envelope genes, desmosomal genes, transcription factors genes, gamma secretase activity genes, sarcoplasmic reticulum genes (Fig. 4; schematic of cardiomyocyte depicting genes associated with non-syndromic familial DCM, in their approximate subcellular context), and for other cardiac genes causally linked with DCM (Hershberger et al. 2013; Pérez-Serra et al. 2016). Z-disk gene variants exhibit remarkable cardiac phenotypic variability, which might also be the case for TTNtvZ variants. Of note are DCM-susceptible Z-disk genes, which are also associated with HCM phenotypes and vice versa (Hassel et al. 2009; Wang et al. 2010b). The varied mechanisms underlying the phenotypic outcome of the Z-disk mutational spectrum are not well understood. On a mechanical basis, the sarcomeric Z-disk functions as a force sensor, integrates and processes biochemical signals, and mutations affecting this structure are, therefore, involved in the maladaptation to biomechanical stress. Various hypotheses have been developed to understand the underlying molecular mechanisms, one of which supports the view that mutations loosening sarcomeres lead to DCM, while other mutations causing an increase in stiffness lead to maladaptive hypertrophy via decreased and increased calcium sensitivity (HCM). In light of this, a subset of mutations in force-generating domains of myofilament proteins were shown to be causes of HCM, while mutations in force-transmitting domains of myofilament proteins were shown to be causes of DCM (Olson et al. 1998, 2000).

In this context, a study by Walsh et al. (2016) re-assessed Mendelian inheritance in 7855 cardiomyopathy cases and, contrary to the increasing number of rare variants being linked to DCM, challenged the pathogenicity of some of the published rare variants (truncating/non-truncating). This criticism also included the nomenclature of TTN nonsense variants as “TTNtv”, as this might be misleading unless truncations are proven by protein gels (Shih et al. 2016). These reports raised questions on the pathogenicity of published truncating variants, especially when the conclusions are not supported by strong data such as large clinical cohorts, background frequencies in population cohorts, and mechanistic/animal studies. It is, therefore, important not only to identify and classify rare variants but also to assess them in the light of disease-causing mechanisms, e.g., whether there is a dominant negative mutation activating (hypercontractile) or deactivating (hypocontractile) or which features are imparted by haploinsufficient mutations. Further, variants reported as pathogenic but without strong evidence of segregation might be regarded as modifier genes or more parsimoniously regarded as being phenotypically silent.

## TTN missense variants

High NGS utility has also led to the identification of a vast number of missense variants in DCM-linked genes, such as desmosomal, sarcomeric, cytoskeletal, nuclear envelope, and ion channels, as well as in genes with minor frequency linked to DCM (Pérez-Serra et al. 2016). Similar to TTNtv, missense variants were also non-uniformly distributed over the length of TTN and were over-represented in A-band TTN (Begay et al. 2015). On the phenotypic levels, no differences (event-free survival from cardiovascular death/transplant) were observed between groups carrying likely pathogenic, possibly pathogenic, and non-carriers. However, careful analysis within the carriers revealed an interesting trend of lower left ventricular ejection fraction, with variants moving from Z-disk towards A-band and lower ejection fraction with double or compound mutations compared to those with single variants (Begay et al. 2015). Based on detailed bioinformatics analysis, the study of Begay et al. (2015) reported 12.6% of TTN missense variants being severe in 27.6% of DCM cases (although the severity of these variants is not supported by segregation analysis), which were enriched in the A-band and which depicts their potential pathogenicity and biological role. In the study by Merlo et al. (2013), 24 missense variants were observed in a sarcomeric panel of genes including TTN in DCM patients presenting with severe phenotypes, including a high frequency of ventricular arrhythmias and high incidence of cardiovascular events in comparison to non-carrier DCM patients. Although these studies support the pathogenicity of TTN missense variants in DCM, in general it is more often the case that missense variants are over-extrapolated for their pathogenicity (Walsh et al. 2016). This conservative/stringent statement highlights the fact that missense variants could be regarded as possible misinterpretable entities, and should be considered carefully for their pathogenicity, e.g., segregation within families, ability to affect the protein stability/alter protein binding, population frequency, etc., before any conclusions can be drawn about them. This has been highlighted in a recent article, where the investigators showed 62 missense variants in 35.2% of DCM patients (*n* = 176) included in the study compared to 187 missense variants found in 37.2% (*n* = 187) of the control population (1000 Genome Europeans) (Dalin et al. 2017). Their findings truly reflect the need to re-characterize the role of missense variants in DCM.

With recent advancements in gene editing technologies such as clustered regularly interspaced short palindromic repeats and Cas9 nucleases (Crispr/Cas9), it is more feasible to introduce truncating variants (nonsense, frameshift, splice site) in tissue-restricted manners as to substantially aid in understanding the pathogenic mechanisms of these variants. In this regard, Deo (2016) introduced TTNtv in zebrafish proximal (N-terminus) and distal (C-terminus) ends of TTN. The introduction of these TTNtv in constitutive exons was instrumental in identifying the location of the internal promoter named Cronos and implicating this region with higher sensitivity towards TTNtv (Zou et al. 2015). Applying similar approaches to introduce proximal and distal TTNtv in rat genomes using zinc finger nuclease-mediated gene targeting, Schafer et al. (2016) demonstrated NMD of these truncating alleles, implying an underlying haploinsufficient mechanism in the pathogenicity of TTNtv. These studies highlight the power of modern gene editing techniques in gaining insight into molecular mechanisms underlying TTNtv and other variants.

## Outlook and future perspective

In summary, our current understanding on truncating and non-truncating variants in DCM and other cardiac phenotypes needs to be explored beyond simple genetic events and on the functional level. The pathogenesis of DCM needs to be addressed, in particular, beyond the explanation of haploinsufficient and dominant negative lesions. Disease-causing mutations in DCM might also be addressed in the epigenetic context. Do truncating variants in different TTN domains and/or bands dictate specific epigenetic lesions? Is there a differential epigenetic sensitivity towards haploinsufficiency or dominant negative TTNtv? How do environmental stressors shape the DCM genome and epigenome in patients with genotype-positive/phenotype-negative individuals and vice versa. The limited number of genotype-positive DCM patients presenting with the spectrum of specific cardiac phenotypes and even more limited patients with one particular mutation are the major obstacles to confidently assigning the risk carried by each variant. On the other hand, it is possible that patients with variable titinopathies could present with converging patterns of epigenetic lesions, which might aid in the development and implementation of epigenotype-specific treatment regimens (i.e., epidrugs) in the management of DCM.

Another important issue is to identify to which extent TTNtv contribute to the phenotype in the presence of other disease-causing mutations. Therefore, in order to establish the pathogenicity of rare variants, further studies should focus on prospective longitudinal population cohorts without apparent overt cardiac phenotypes needing to be established if the occurrence of cardiac events (if any) is significant in pre-symptomatic genotype-positive individuals. This can help the geneticist in counseling such individuals, or in the design of screening programs which can help to identify further family members at risk, and, finally, develop prophylactic managements (all of which could possibly be initiated before the onset of disease). However, considerable challenges lie ahead, including the integration of masses of genetic information and their interpretation in the biological context. This will also comprise building standard algorithms for comprehensive diagnostic workups in the evaluation and management of inherited cardiac disease.

Given the gigantic size of TTN, it is expected that the TTNtv landscape of DCM will increase in much higher numbers than currently known. As discussed, truncating changes are likely to be pathogenic; however, the mechanism of pathogenicity is probably less well understood. Recent functional studies highlighting the position-independent toxicity of TTNtv raise further questions on dominant negative modes of action than previously envisaged. If evidence is generalized to broader populations of TTNtv, then the preponderance of TTNtv in the A-band requires further explanation.

## Abbreviations

Crispr: Clustered regularly interspaced short palindromic repeats
CVD: Cardiovascular disease
DCM: Dilated cardiomyopathy
ExAC: Exome Aggregation Consortium
eGFP: Enhanced green fluorescent protein
ENU: Ethylnitrosourea
FINmaj: Finnish founder mutation
GWAS: Genome-wide association studies
HCM: Hypertrophic cardiomyopathy
HF: Heart failure
LV: Left ventricle
NGS: Next-generation sequencing
NMD: Nonsense-mediated mRNA decay
PEVK: Pro-Glu-Val-Lys titin domain
TTN: Titin
TTNtv: Titin-truncating variants
TTNtvA: TTNtv at the A-band titin
TTNtvZ: TTNtv at Z-disk titin
